# Technology-based interventions for health challenges older women face amid COVID-19: a systematic review protocol

**DOI:** 10.1186/s13643-022-02150-9

**Published:** 2022-12-13

**Authors:** Zhaohui Su, Ali Cheshmehzangi, Barry L. Bentley, Dean McDonnell, Sabina Šegalo, Junaid Ahmad, Hengcai Chen, Lori Ann Terjesen, Emme Lopez, Shelly Wagers, Feng Shi, Jaffar Abbas, Caifeng Wang, Yuyang Cai, Yu-Tao Xiang, Claudimar Pereira da Veiga

**Affiliations:** 1 School of Public Health, Institute for Human Rights, Southeast University, Nanjing, 210009 China; 2 Faculty of Science and Engineering, University of Nottingham Ningbo China, Ningbo, Zhejiang, 315100 China; 3 Network for Education and Research on Peace and Sustainability (NERPS), Hiroshima University, Hiroshima, 739-8530 Japan; 4 Cardiff School of Technologies, Cardiff Metropolitan University, Cardiff, UK; 5 Collaboration for the Advancement of Sustainable Medical Innovation, University College London, London, UK; 6 Department of Humanities, South East Technological University, Carlow, R93 V960 Ireland; 7 Faculty of Health Studies, University of Sarajevo, 71000 Sarajevo, Bosnia and Herzegovina; 8 Rufaidah Nursing College, Peshawar, Pakistan; 9 Faculty of Science and Engineering, University of Nottingham Ningbo China, Ningbo, 315100 China; 10 National Women’s History Museum, Alexandria, USA; 11 UT Health San Antonio, San Antonio, USA; 12 Department of Criminology, University of South Florida St. Petersburg, St. Petersburg, USA; 13 Department of Research and Development, Shanghai United Imaging Intelligence, Shanghai, China; 14 Antai College of Economics and Management, and School of Media and Communication, Shanghai Jiao Tong University, Shanghai, 200240 China; 15 Department of Environmental Health, School of Public Health, School of Nursing, Shanghai Jiao Tong University School of Medicine, 280 South Chongqing Road, Shanghai, 200025 China; 16 School of Public Health, China Institute for Urban Governance, Shanghai Jiao Tong University School of Medicine, Shanghai, 200025 China; 17 Unit of Psychiatry, Department of Public Health and Medicinal Administration, Institute of Translational Medicine, Faculty of Health Sciences, Centre for Cognitive and Brain Sciences, Institute of Advanced Studies in Humanities and Social Sciences, University of Macau, Macao SAR, China; 18 Fundação Dom Cabral – FDC, Av. Princesa Diana, 760 Alphaville, Lagoa dos Ingleses, Nova Lima, MG 34018-006 Brazil

**Keywords:** COVID-19, Older people, Women, Technology-based interventions, Health disparities, Ageing

## Abstract

**Background:**

Pandemics, such as COVID-19, are dangerous and socially disruptive. Though no one is immune to COVID-19, older persons often bear the brunt of its consequences. This is particularly true for older women, as they often face more pronounced health challenges relative to other segments in society, including complex care needs, insufficient care provisions, mental illness, neglect, and increased domestic abuse. To further compound the situation, because protective measures like lockdowns can result in unintended consequences, many health services older women depend on can become disrupted or discontinued amid pandemics. While technology-based interventions have the potential to provide near-time, location-free, and virtually accessible care, there is a dearth of systematic insights into this mode of care in the literature. To bridge the research gaps, this investigation aims to examine the characteristics and effectiveness of technology-based interventions that could address health challenges older women face amid COVID-19.

**Methods:**

A systematic review of randomized trials reporting on technology-based interventions for older women (≥65 years) during COVID-19 will be conducted. The databases of Web of Science, ScienceDirect, PubMed/MEDLINE, PsycINFO, CINAHL, and Scopus will be searched. Retrieved citations will be screened independently by at least two reviewers against the eligibility criteria. Included studies will be assessed using the Cochrane ROB-2 tool. Data will be extracted independently by the reviewers. Where possible, meta-analyses will be performed on relevant study outcomes and analysed via odds ratios on the dichotomized outcomes. Where applicable, heterogeneity will be measured using the Cochrane Q test, and publication bias will be assessed via funnel plots and Egger’s regression test.

**Discussion:**

Technology has the potential to transform healthcare for the better. To help society better safeguard vulnerable populations’ health and quality of life, this investigation sets out to gauge the state-of-the-art development of technology-based interventions tailored to the health challenges older women face amid COVID-19. In light of the growing prevalence of population ageing and the inevitability of infectious disease outbreaks, greater research efforts are needed to ensure the timely inception and effective implementation of technology-based health solutions for vulnerable populations like older women, amid public health crises like COVID-19 and beyond.

**Systematic review registration:**

PROSPERO CRD42020194003

## Background

COVID-19 has proven to be a dangerous and socially disruptive disease [1–3]. It is also fast evolving, producing or perpetuating a cascade of crises, ranging from the rising global mental health epidemic to surging infections amongst refugees from the conflict in Ukraine [4–9]. As of December 9, 2022, official records show that the pandemic is responsible for around 650 million infections and 7 million deaths across the world [10]. Though already sobering, these numbers are widely deemed as merely a portion of the true toll of the pandemic [1–3]. While no one is immune to COVID-19, older persons—individuals aged 65 and over—often bear the brunt of its consequences. In an analysis conducted by the World Health Organization, for instance, researchers estimated that approximately 82% of worldwide pandemic-related excess deaths occurred amongst older persons [11]. In addition to being susceptible to COVID-19 infections, hospitalizations, and deaths, growing evidence shows that older age and female gender are two risk factors for prolonged post-COVID syndromes [12–14], such as fatigue and cognitive impairment. As ageing increases susceptibility to infections in older persons, while also reducing their regenerative capacity, developments are sorely needed to treat the underlying pathologies of ageing [15–17]. Uniquely for women, the complex interplay between social and biological factors is also likely to play a role in their susceptibility to COVID-19, as well as the scale, scope, and severity of the health challenges they face.

Firstly, compared to their male counterparts, older women are often more likely to suffer from certain types of poor health. Research shows, for instance, that the prevalence of frailty and prefrailty amongst older women (44.8 frailty and 173.2 prefrailty cases per 1000 individuals) is significantly more severe compared to men (24.3 frailty and 129.0 prefrailty cases per 1000 individuals) [18]. Compared to older men, older women are also more likely to shoulder mental health stressors or disorders [19]. In a longitudinal study conducted in the Netherlands, researchers found that older women are 30% more likely to face depressive symptoms when compared to older men [20]. Further complicating the situation, a growing body of evidence shows that the pandemic has increased older women’s rates of mental health stress [21–23]. Secondly, older women face distinct health challenges arising from gender-based neglect and violence. It is estimated that approximately one in every six older women experiences abuse and/or neglect across the globe [24]. In an analysis of 3354 community-dwelling older women in the United States (US), for example, researchers found that 14% of the participants were physically and/or sexually abused [25]. Thirdly, older women are more likely to face systemic health disparities than older men. Due to the diseases or disabilities they face, a number of older women may struggle to address their health needs and daily activities [26–29].

Poor access to care, and more complex care needs resulting from longer life expectancy, may partially explain why older women often resort to institutional care in later life, as opposed to remaining in the community and ageing in familiar environments like home. In the US, for instance, pre-pandemic analysis shows that women constitute around 70.2% of the long-term residential care population [30]. Due to the shifting impacts of the pandemic, there are limited up-to-date data on how many older women are living in nursing homes. What is clear, though, is that the care and services provided by these facilities are often suboptimal. Across the pandemic, recurring investigations show that many older persons, especially those who have cognitive or physical impairments, are often being “abandoned to die” in nursing homes [31–33]. These factors combined, overall, reveal the degree of health disparities older persons face amid COVID-19, particularly amongst women. Despite their pronounced need for timely and effective interventions, there is a shortage of research on health solutions that are tailored to this population, especially agile and versatile ones, such as technology-based interventions, that could circumvent the unintended consequences posed by pandemic-related lockdowns or other physical distancing measures.

Technology-based interventions are defined as the adoption and application of technological tools or techniques in the design, development, and delivery of health solutions to the intended users [34], such as technology-based health interventions using readily available devices (e.g. smartphones, mobile sensors, or gaming consoles) to manage, support, or deliver accessible and affordable health solutions to persons in need of healthcare services [34]. Public health policies, such as lockdowns and social distancing, are being used to disrupt physical contact and limit interactions, with the aim of reducing virus transmission. However, because human interaction is a significant social lifeline for individuals, particularly older persons with limited mobility, the need for technology-based interventions is more pronounced. Although technology-based interventions have great potential to address health challenges older women face amid pandemics, such as COVID-19, overall, there is a shortage of evidence in the literature. Therefore, to bridge the research gap, this investigation examines the characteristics and effectiveness of technology-based interventions to address health challenges older women face amid social isolation and COVID-19.

## Methods

Following best practices, the International Prospective Register of Systematic Reviews database or PROSPERO (CRD42020194003) was used for study registration, and the Preferred Reporting Items for Systematic reviews and Meta-Analyses (PRISMA) was selected for the literature searching and screening process [35]. These steps were taken to ensure that the current review is in line with recommended practice, as well as to boost research transparency and the replicability of review findings for the research field [36–41].

### Eligibility criteria

The aim of the current investigation is to research the characteristics and effectiveness of technology-based interventions that can be used to address health challenges faced by older women amid COVID-19 and future pandemics. Considering eligibility criteria, having a clear and concrete understanding of the population and the specific research problem will help identify components of the PICOS framework applied to this study: namely the Population, Intervention, Comparison, Outcome, and Study design [42]. The eligibility criteria are considered from two perspectives: inclusion and exclusion criteria. A list of inclusion criteria can be found in Table 1, while, overall, studies will be excluded if they (1) do not focus on women 65 years and over (e.g. middle-aged men or women), (2) do not focus on health challenges older women face post-COVID (e.g. diseases in children), (3) do not focus on technology-based interventions (e.g. in-person mental health consultations), (4) do not report detailed information of the interventions studied (e.g. characteristics of the interventions), (5) are not conducted in the context of COVID-19, and (6) do not report empirical research findings (e.g. effects of the interventions, such as changes in the study populations’ mental health).

**Table 1:** Study inclusion criteria

Category	Criteria
Population	Women who are 65 years and older (≥50% of the whole population studied), with or without health conditions
Intervention	Technology-based interventions (i.e. examining the purpose and application of the intervention, intervention exposure, outcome variables assessed/measured, and whether the design of the intervention material is tailored to women or the epidemic/pandemic context)
Comparison	Non-technology-based interventions
Outcome	Reported characteristics and effectiveness of the technology-based interventions. For instance, if the interventions were tailored to improve participants’ mental health amid COVID-19, then our team will record the mechanisms of the interventions that aim to elevate the participants’ mental health, and the corresponding measures that the reviewed studies adopted to gauge the changes in the respondents’ mental health (e.g. anxiety, depression, post-traumatic stress disorder). Below are more specific examples: • Changes in physical health (e.g. walking distance, fall frequencies, frailty symptoms.) • Changes in mental health (e.g. anxiety, depression, post-traumatic stress disorder, suicidal attempts) • Changes in quality of life (e.g. life satisfaction, happiness, perceived health status)
Research design	Randomized controlled trials (RCTs) and quasi-RCTs

### Search strategy

Our key search terms will be centred on three concepts: older women, technology-based interventions, and COVID-19, and developed in consultation with an academic librarian. An initial PubMed/MEDLINE search string using MeSH and key terms is included in Table 2. Search strings will be subsequently applied to Web of Science, ScienceDirect, PubMed/MEDLINE, PsycINFO, CINAHL, and Scopus databases. The search will be conducted in August 2023. Drawing insights from previous studies [43, 44], in addition to database searches, we will also manually search reference lists of the included articles to identify additional eligible papers.

**Table 2:** Initial PubMed/MEDLINE search string

Concept	Search string
Older women	“older women”[MeSH] OR “older women”[TIAB] OR “older woman”[TIAB] OR “older women”[TIAB] OR “older female*”[MeSH] OR “older female*”[TIAB] “elder women”[MeSH] OR “elder women”[TIAB] OR “elder woman”[TIAB] OR “elder women”[TIAB] OR “elder female*”[MeSH] OR “elder female*”[TIAB]
Technology-based interventions	“technology”[MeSH] OR “technology”[TIAB] OR “eHealth”[TIAB] OR “telemedicine”[MeSH] OR “Artificial Intelligence” [MeSH] OR “telemedicine”[TIAB] OR “tele-medicine”[MeSH] OR “tele-medicine”[TIAB] OR “telehealth”[TIAB] OR “tele-health”[TIAB] OR “connected health”[TIAB] OR “digital health”[TIAB] OR “mHealth”[TIAB] OR “mobile health”[TIAB]
COVID-19	“pandemics”[MeSH] OR “pandemic”[TIAB] OR “pandemics”[TIAB] OR “epidemics”[MeSH] OR “epidemic”[TIAB] OR “epidemics”[TIAB] OR “SARS Virus”[MeSH] OR SARS[TIAB] OR “Coronavirus Infections”[Mesh:NoExp] OR “COVID-19”[Supplementary Concept] OR “severe acute respiratory syndrome”[TIAB] OR “Severe Acute Respiratory Syndrome”[MeSH] OR “covid”[TIAB] OR (novel[TIAB] AND coronavirus[TIAB]) OR ((coronavirus[TIAB] OR “corona virus”[TIAB] OR coronavirinae[TIAB] OR coronaviridae[TIAB] OR betacoronavirus[TIAB] OR covid19[TIAB] OR “covid 19”[TIAB] OR nCoV[TIAB] OR “CoV 2”[TIAB] OR CoV2[TIAB] OR sarscov2[TIAB] OR 2019nCoV[TIAB] OR “novel CoV”[TIAB] AND (“severe acute respiratory”[TIAB] OR pneumonia[TIAB]) AND (outbreak[TIAB])) OR “Coronavirus”[MeSH] OR “Coronavirus Infections”[MeSH] OR “COVID-19”[Supplementary Concept] OR “severe acute respiratory syndrome coronavirus 2”[Supplementary Concept] OR “Betacoronavirus”[MeSH])

### Study selection

Upon search completion, citations will be uploaded to Rayyan [45], with duplicates removed. Principal reviewers will screen titles and abstracts against the selection and exclusion criteria independently. Reasons for exclusion will be recorded and reported in detail in the PRISMA flowchart. When the initial pool of records has been identified, the principal reviewers will compare and contrast their screening and selection results to evaluate the scale and scope of any discrepancies. If differences of opinion persist after discussions between the principal reviewers, group meetings will be held to ensure the finalized database is agreed upon by all authors. Full-text articles will then be obtained for detailed review. Articles excluded in this process will also be carefully recorded and reviewed, especially if there is a discrepancy between the principal reviewers. Prolonged discrepancies will be resolved via virtual or in-person group discussions. After the final article pool is identified, references listed in these papers will be surveyed to determine if additional studies also meet the eligibility criteria.

### Study quality assessment

The quality of eligible studies will be evaluated with the guidance of the Revised Cochrane Risk-Of-Bias tool for randomized trials (ROB-2) [46]. In the context of systematic review and meta-analysis studies, bias can be understood as “a systematic deviation from the effect of intervention that would be observed in a large randomized trial without any flaws” [46]. The ROB-2 framework has the following segments: Risk of bias arising from the randomization process, risk of bias due to deviations from the intended interventions (effect of assignment/adhering to intervention), missing outcome data, risk of bias in the measurement of the outcome, risk of bias in the selection of the reported result, and overall risk of bias [47]. The independent reviewers will focus on judging each segment in terms of potential material risks that could have had a noticeable impact on the study outcomes, and subsequently categorize the risk levels as the following: “low risk of bias”, “some concerns,” or “high risk of bias” [46, 47]. The ROB-2 assessment template will be adopted to facilitate the review process [47].

### Data extraction and synthesis

A comprehensive list of data will be extracted, including study characteristics (e.g. country of origin, study methods, and research purpose), sample characteristics (e.g. age, race/ethnicity, and disease history), interventions evaluated (e.g. intervention stimuli, intervention exposure, and the use of technology), outcome variables assessed (e.g. before-after health outcome changes), and principal research findings. Data extraction will be conducted by the principal reviewers independently. Descriptive analysis will be used to identify any salient patterns amongst included articles (e.g. country of origin distribution), whereas narrative synthesis will be adopted to investigate the characteristics and effects of the interventions. Where possible, meta-analyses will be performed on relevant study outcomes (e.g. changes in mental health) to estimate intervention efficacy; odds ratio analysis will be performed on dichotomized outcomes and mean difference for continuous outcomes, or standardized mean difference for similar outcomes measured in different ways, with 95% confidence intervals; heterogeneity will be measured using Cochrane’s Q test. Publication bias will be addressed via funnel plots and Egger’s regression test. If meta-analysis is not feasible, we will report the results narratively.

## Discussion

**Fig. 1:**
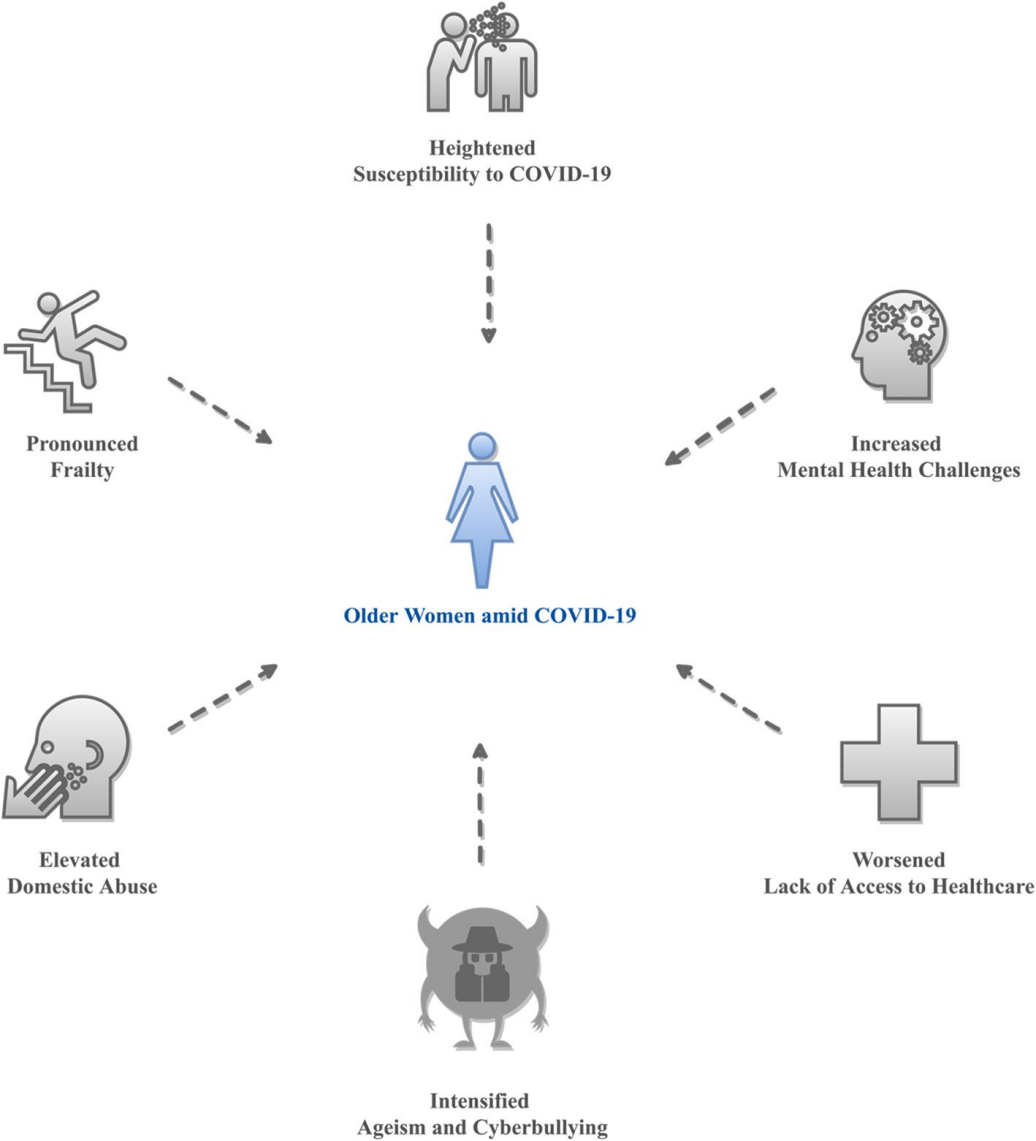
Main health challenges older women face amid COVID-19

Technology has the potential to transform healthcare for the better [48–54]. To help society better safeguard vulnerable populations’ health and quality of life, this research sets out to investigate the characteristics and effectiveness of technology-based interventions that can be used to address health challenges older women face amid COVID-19. To our knowledge, this is one of the first studies that focused on identifying state-of-the-art technology-based solutions that are tailored to health challenges older women shoulder during pandemics. COVID-19, along with its resultant crises, has both introduced and intensified threats to older women’s health and quality of life, ranging from heightened risks of COVID-19 infections and deaths, increased elder neglect and abuse, increased gender-based violence and discrimination, additional mental health challenges, and curtailed or cancelled access to health services (see Fig. 1) [53, 55–57]. As women are often charged with formal or informal caregiving roles and responsibilities [58], failing to address the health challenges they face could not only compromise their health and wellbeing, but also those in their broader communities.

By identifying the characteristics and effectiveness of technology-based interventions available amid the pandemic, the findings of this investigation have the potential to offer timely solutions to alleviate the threats that undermine older women’s health and quality of life. Based on effective technology-based solutions identified from the literature, insights from this systematic review can also help researchers better design, develop, and deploy technology-based interventions to support older persons through future public health crises of COVID-19’s scale. In other words, a comprehensive understanding of the characteristics and effectiveness of technology-based health solutions available to older women in the context of pandemics can also help researchers discover areas of improvement regarding intervention design and development for future pandemics (e.g. antimicrobial resistance). In the current and future investigations, we will pay special attention to issues such as (1) whether and to what extent technology-based interventions have mechanisms that could protect older women’s privacy and security (e.g. whether the user data would be shared with third parties) and (2) whether and to what extent these interventions are developed from older women’s perspectives (e.g. whether the interventions are dependent on expensive electronics or high-speed Internet connectivity).

Technology, regardless of how advanced it might be, is not immune to shortcomings [48, 59–62]. It is important to not only understand technology-based interventions’ power and promise in safeguarding older women’s health and wellbeing amid crises like COVID-19, but also the responsibility and accountability these critical solutions shoulder—or fail to shoulder—to ensure health services that aim to help do not incur harm [63]. Having a connected and comprehensive understanding of technology-based interventions’ ability to improve health outcomes, and the potential to introduce unwanted consequences, could ensure their healthy and sustainable development, and in turn, their long-term capability to protect and promote the health and wellbeing of older persons, and particularly women. Overall, in light of the changing demographics of an ageing population, and the inevitability of infectious disease outbreaks [4], greater research efforts are needed to ensure the timely inception and effective implementation of technology-based health solutions for vulnerable populations like older women, amid crises like COVID-19 and beyond.

## Data Availability

Data are available upon reasonable request.

## Abbreviations

COVID-19: Coronavirus Disease 2019
MeSH: Medical Subject Heading
PICOS: Population, Intervention, Comparison, Outcome, and Study design
PRISMA: The Preferred Reporting Items for Systematic reviews and Meta-Analyses
PROSPERO: International Prospective Register of Systematic Reviews database
US: The United States
RCT: Randomized Controlled Trial
ROB-2: Revised Risk Of Bias tool
TIAB: Title or Abstract

## References

[1] The Economist (2022). Tracking covid-19 excess deaths across countries.

[2] World Health Organization (2022). The true death toll of COVID-19: estimating global excess mortality.

[3] Wang H (2022). Estimating excess mortality due to the COVID-19 pandemic: a systematic analysis of COVID-19-related mortality, 2020–21. Lancet.

[4] Su Z (2022). Public health crises and Ukrainian refugees. Brain Behav Immun.

[5] Santomauro DF (2021). Global prevalence and burden of depressive and anxiety disorders in 204 countries and territories in 2020 due to the COVID-19 pandemic. Lancet.

[6] Su Z (2021). Mental health consequences of COVID-19 media coverage: the need for effective crisis communication practices. Glob Health.

[7] Su Z (2022). Mind the “vaccine fatigue”. Front Immunol.

[8] Su Z (2022). Mind the “worry fatigue” amid Omicron scares. Brain Behav Immun.

[9] Su Z (2022). Media-induced war trauma amid conflicts in Ukraine. Perspect Psychol Sci.

[10] Our World in Data (2022). Coronavirus pandemic (COVID-19).

[11] World Health Organization (2022). 14.9 million excess deaths associated with the COVID-19 pandemic in 2020 and 2021.

[12] Evans RA (2022). Clinical characteristics with inflammation profiling of long COVID and association with 1-year recovery following hospitalisation in the UK: a prospective observational study. Lancet Respir Med.

[13] Antonelli M (2022). Risk factors and disease profile of post-vaccination SARS-CoV-2 infection in UK users of the COVID Symptom Study app: a prospective, community-based, nested, case-control study. Lancet Infect Dis.

[14] Sigfrid L (2021). Long Covid in adults discharged from UK hospitals after Covid-19: A prospective, multicentre cohort study using the ISARIC WHO Clinical Characterisation Protocol. Lancet Reg Health Eur.

[15] Calimport SRG (2019). To help aging populations, classify organismal senescence. Science.

[16] Sills J (2020). The inherent challenges of classifying senescence—Response. Science.

[17] Calimport SRG, Bentley BL (2019). Aging classified as a cause of disease in ICD-11. Rejuvenation Res.

[18] Ofori-Asenso R (2019). Global incidence of frailty and prefrailty among community-dwelling older adults: a systematic review and meta-analysis. JAMA Netw Open.

[19] Amir-Behghadami M (2020). Psychometric properties of the Iranian version of self-care ability scale for the elderly. BMC Geriatr.

[20] Sialino LD (2020). Sex differences in mental health among older adults: Investigating time trends and possible risk groups with regard to age, educational level and ethnicity. Aging Ment Health.

[21] Pierce M (2020). Mental health before and during the COVID-19 pandemic: a longitudinal probability sample survey of the UK population. Lancet Psychiatry.

[22] Barber SJ, Kim H (2021). COVID-19 worries and behavior changes in older and younger men and women. J Gerontol: Series B.

[23] González-Sanguino C (2020). Mental health consequences during the initial stage of the 2020 Coronavirus pandemic (COVID-19) in Spain. Brain Behav Immun.

[24] Yon Y (2019). The prevalence of self-reported elder abuse among older women in community settings: a systematic review and meta-analysis. Trauma Violence Abuse.

[25] Cook JM (2013). Prevalence of physical and sexual assault and mental health disorders in older women: findings from a nationally representative sample. Am J Geriatr Psychiatry.

[26] Tabrizi JS (2018). Self-care ability of older people living in urban areas of Northwestern Iran. Iran J Public Health.

[27] Manuel JI (2018). Racial/ethnic and gender disparities in health care use and access. Health Serv Res.

[28] Zhang J (2020). Gender difference in the association of frailty and health care utilization among Chinese older adults: results from a population-based study. Aging Clin Exp Res.

[29] Carrero JJ (2018). Sex and gender disparities in the epidemiology and outcomes of chronic kidney disease. Nat Rev Nephrol.

[30] U.S. Department of Health And Human Services, Long-term care providers and services users in the United States, 2015–2016, in Vital and Health Statistics. 2019, Centers for Disease Control and Prevention,,.

[31] Amnesty International (2021). UK: Older people in care homes abandoned to die amid government failures during COVID-19 pandemic.

[32] Trabucchi M, de Leo D (2021). Nursing homes or abandoned castles: COVID-19 in Italy. Lancet Psychiatry.

[33] Mahase E (2021). Covid-19: Neglect was one of biggest killers in care homes during pandemic, report finds. BMJ.

[34] Su Z (2020). Understanding technology-based interventions for caregivers of cancer patients: a systematic review-based concept analysis. J Med Internet Res.

[35] Moher D (2009). Preferred Reporting Items for Systematic Reviews and Meta-Analyses: the PRISMA statement. PLoS Med.

[36] Stewart L, Moher D, Shekelle P (2012). Why prospective registration of systematic reviews makes sense. Syst Rev.

[37] Chang SM, Slutsky J (2012). Debunking myths of protocol registration. Syst Rev.

[38] Xu C (2019). Protocol registration or development may benefit the design, conduct and reporting of dose-response meta-analysis: Empirical evidence from a literature survey. BMC Med Res Methodol.

[39] dos Santos MBF (2020). Protocol registration improves reporting quality of systematic reviews in dentistry. BMC Med Res Methodol.

[40] Shokraneh F (2019). Reproducibility and replicability of systematic reviews. World J Meta-Anal.

[41] Ramstrand N (2019). Promoting quality and transparency in clinical research. Prosthet Orthot Int.

[42] Amir-Behghadami M, Janati A (2020). Population, Intervention, Comparison, Outcomes and Study (PICOS) design as a framework to formulate eligibility criteria in systematic reviews. Emerg Med J.

[43] Amir Behghadami M (2019). Developing and validating an instrument to assess non-hospital health centers' preparedness to provide initial emergency care: a study protocol. BMJ Open.

[44] Behghadami MA (2019). Assessing preparedness of non-hospital health centers to provide primary emergency care; a systematic review. Bull Emerg Trauma.

[45] Ouzzani M (2016). Rayyan — a web and mobile app for systematic reviews. Syst Rev.

[46] Sterne JAC (2019). RoB 2: a revised tool for assessing risk of bias in randomised trials. BMJ.

[47] Risk of bias tools (2019). Current version of RoB 2.

[48] Su Z (2022). Technology-based mental health interventions for domestic violence victims amid COVID-19. Int J Environ Res Public Health.

[49] Su Z, Chengbo Z, Mackert M (2019). Understanding the influenza vaccine as a consumer health technology: a structural equation model of motivation, behavioral expectation, and vaccine adoption. J Commun Healthcare.

[50] Su Z (2021). Technology-based interventions for cancer caregivers: a concept analysis. JMIR Cancer.

[51] Su Z (2021). Technology-based health solutions for cancer caregivers to better shoulder the impact of COVID-19: a systematic review protocol. Syst Rev.

[52] Su Z (2021). Technology-based interventions for nursing home residents: a systematic review protocol. BMJ Open.

[53] Yang Y (2020). Mental health services for older adults in China during the COVID-19 outbreak. Lancet Psychiatry.

[54] Su Z (2021). Addressing Biodisaster X threats with artificial intelligence and 6G technologies: literature review and critical insights. J Med Internet Res.

[55] Rosenthal AE (2022). The past and future of gender nondiscrimination policy under the affordable care act. Am J Prev Med.

[56] Su Z (2021). Gender inequality and health disparity amid COVID-19. Nurs Outlook.

[57] Son Y-J (2021). Gender differences in the impact of frailty on 90-day hospital readmission in heart failure patients: a retrospective cohort study. Eur J Cardiovasc Nurs.

[58] Organisation for Economic Co-operation and Development (2021). Caregiving in crisis: gender inequality in paid and unpaid work during COVID-19.

[59] Kutzner F, Fiedler K (2017). Stereotypes as pseudocontingencies. Eur Rev Soc Psychol.

[60] Su Z (2021). Vaccines are not yet a silver bullet: the imperative of continued communication about the importance of COVID-19 safety measures. Brain Behav Immun Health.

[61] Su Z (2020). A race for a better understanding of COVID-19 vaccine non-adopters. Brain Behav Immun Health.

[62] Mozilla (2022). Top mental health and prayer apps fail spectacularly at privacy, security.

[63] Su Z (2021). Rigorous policy-making amid COVID-19 and beyond: literature review and critical insights. Int J Environ Res Public Health.

